# Multi-Omics and Network-Based Drug Repurposing for Septic Cardiomyopathy

**DOI:** 10.3390/ph18010043

**Published:** 2025-01-02

**Authors:** Pei-Pei Liu, Xin-Yue Yu, Qing-Qing Pan, Jia-Jun Ren, Yu-Xuan Han, Kai Zhang, Yan Wang, Yin Huang, Tao Ban

**Affiliations:** 1 Department of Pharmacology, College of Pharmacy, Harbin Medical University, Harbin 150081, China; 2 Key Laboratory of Drug Quality Control and Pharmacovigilance, Ministry of Education, China Pharmaceutical University, Nanjing 210009, China; 3 Department of Pharmaceutical Analysis, School of Pharmacy, China Pharmaceutical University, Nanjing 210009, China; 4 Department of Critical Care Medicine, Nanjing Drum Tower Hospital, Clinical College, Nanjing Medical University, Nanjing 210008, China; 5 State Key Laboratory of Frigid Zone Cardiovascular Diseases, Ministry of Science and Technology, Harbin Medical University, Harbin 150081, China; 6 Key Laboratory of Cardiovascular Research, Ministry of Education, Harbin Medical University, Harbin 150081, China

**Keywords:** metabolomics, transcriptomics, network medicine, acetaminophen, LC-MS

## Abstract

Background/Objectives: Septic cardiomyopathy (SCM) is a severe cardiac complication of sepsis, characterized by cardiac dysfunction with limited effective treatments. This study aimed to identify repurposable drugs for SCM by integrated multi-omics and network analyses. Methods: We generated a mouse model of SCM induced by lipopolysaccharide (LPS) and then obtained comprehensive metabolic and genetic data from SCM mouse hearts using ultra-performance liquid chromatography–tandem mass spectrometry (UPLC–MS/MS) and RNA sequencing (RNA-seq). Using network proximity analysis, we screened for FDA-approved drugs that interact with SCM-associated pathways. Additionally, we tested the cardioprotective effects of two drug candidates in the SCM mouse model and explored their mechanism-of-action in H9c2 cells. Results: Network analysis identified 129 drugs associated with SCM, which were refined to 14 drug candidates based on strong network predictions, proven anti-infective effects, suitability for ICU use, and minimal side effects. Among them, acetaminophen and pyridoxal phosphate significantly improved cardiac function in SCM moues, as demonstrated by the increased ejection fraction (EF) and fractional shortening (FS), and the reduced levels of cardiac injury biomarkers: B-type natriuretic peptide (BNP) and cardiac troponin I (cTn-I). In vitro assays revealed that acetaminophen inhibited prostaglandin synthesis, reducing inflammation, while pyridoxal phosphate restored amino acid balance, supporting cellular function. These findings suggest that both drugs possess protective effects against SCM. Conclusions: This study provides a robust platform for drug repurposing in SCM, identifying acetaminophen and pyridoxal phosphate as promising candidates for clinical translation, with the potential to improve treatment outcomes in septic patients with cardiac complications.

## 1. Introduction

Septic cardiomyopathy (SCM) is an acute cardiac dysfunction that arises in patients with sepsis, a life-threatening organ dysfunction caused by a dysregulated host response to infection [1]. SCM is often clinically characterized by biventricular systolic and diastolic dysfunction, with a particular emphasis on left ventricular impairment [2]. Many studies have demonstrated that SCM patients have worse outcomes compared to those without myocardial dysfunction [3,4,5]. Mortality rates can be as high as 70% in SCM patients [6]. In addition, SCM contributes to prolonged intensive care unit (ICU) stays, higher rates of multi-organ failure, and poor functional recovery after critical illness [7]. The pathogenesis of SCM is complex and multifactorial, involving various cellular and molecular pathways. One of the primary drivers of SCM is the profound immune response elicited by bacterial endotoxins, which leads to the release of a host of inflammatory mediators, including cytokines such as tumor necrosis factor-alpha (TNF-α), interleukin-1β (IL-1β), and interleukin-6 (IL-6) [8]. These mediators not only exacerbate systemic inflammation but also have a direct impact on myocardial cells, impairing their function. Other contributing factors include microvascular dysfunction, oxidative stress, and autonomic dysregulation [9]. Thus, SCM is the result of a synergistic interplay of immune and metabolic processes during sepsis, making its clinical management quite challenging.

Current treatments of SCM center around supportive care, with a focus on managing the underlying sepsis and optimizing cardiovascular function [10]. While numerous therapeutic strategies have been investigated, effective treatments for SCM remain limited. For example, inotropic agents, such as dobutamine and milrinone, are commonly used to improve cardiac contractility in patients with SCM [11]. These drugs increase intracellular cyclic adenosine monophosphate (cAMP) levels, enhancing myocardial contractility. However, the use of inotropes in SCM patients must be carefully balanced, as excessive inotropic stimulation can lead to increased myocardial oxygen demand and further myocardial injury [12]. In addition, vasopressors, particularly norepinephrine, are also frequently employed to counteract the systemic vasodilation associated with sepsis and to maintain adequate perfusion pressure [13]. While effective in raising blood pressure, vasopressors can exacerbate afterload on an already failing heart, potentially worsening myocardial function [14]. Therefore, the development of safe and effective therapies that address the underlying molecular mechanisms of SCM remains a critical unmet need.

In recent years, omics technologies, such as transcriptomics and metabolomics, have provided significant insights into the molecular mechanisms underlying SCM. Studies utilizing transcriptomic and proteomic approaches have revealed the upregulation of genes involved in the inflammatory response (e.g., NF-kappa B signaling pathway, and leukocyte cell–cell adhesion) and pro-inflammatory cytokines (e.g., IL-1β, IL-6, and TNF-α) in heart tissues of animal models [15,16,17]. Metabolomic analyses have also uncovered significant changes in cardiac energy metabolism during sepsis [18,19]. These omics-based studies provide potential targets for therapeutic intervention of SCM. Using these targets, several studies have performed in silico drug repurposing to identify new therapeutic uses in SCM for existing drugs [20,21]. For example, Feng et al. identified the anticancer drug dasatinib as a new therapeutic agent for SCM by analyzing the gene expression profiles taken from the SCM rat hearts [20]. However, these studies lack experimental and clinical validations. Drugs repurposed from other conditions may not adequately address the unique aspects of SCM. Thus, there have been no specific therapies developed for directly targeting SCM yet.

**Figure 1:**
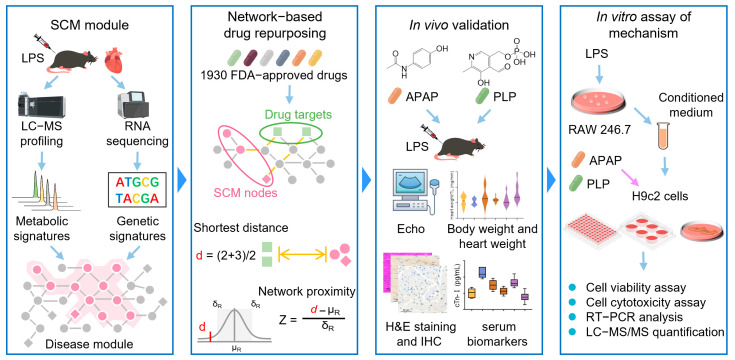
Overall workflow of this study.

In this study, we utilized a systems biology framework that integrated multi-omics and network-based approaches, along with cellular and animal models, to prioritize potential therapeutic agents for SCM from a pool of 1930 drugs approved by the U.S. Food and Drug Administration (FDA). Specifically, we characterized the metabolic and genetic signatures of SCM by analyzing the hearts of lipopolysaccharide (LPS)-induced SCM mice using ultra-performance liquid chromatography–tandem mass spectrometry (UPLC–MS/MS) and RNA sequencing (RNA-seq). We constructed a human metabolite–protein network and found the SCM-associated module within it. We computationally identified some candidates by quantifying the network relationships between each FDA-approved drug and SCM. Network analysis identified 129 drugs associated with SCM, which were refined to 14 drug candidates based on strong network predictions, proven anti-infective effects, suitability for ICU use, and minimal side effects. Finally, we tested the cardioprotective effects of two drug candidates in the SCM mouse model and explored their mechanism-of-action in H9c2 cells (Figure 1).

## 2. Results

### 2.1. LPS-Induced Sepsis Results in the Impairment of Heart Function

**Figure 2:**
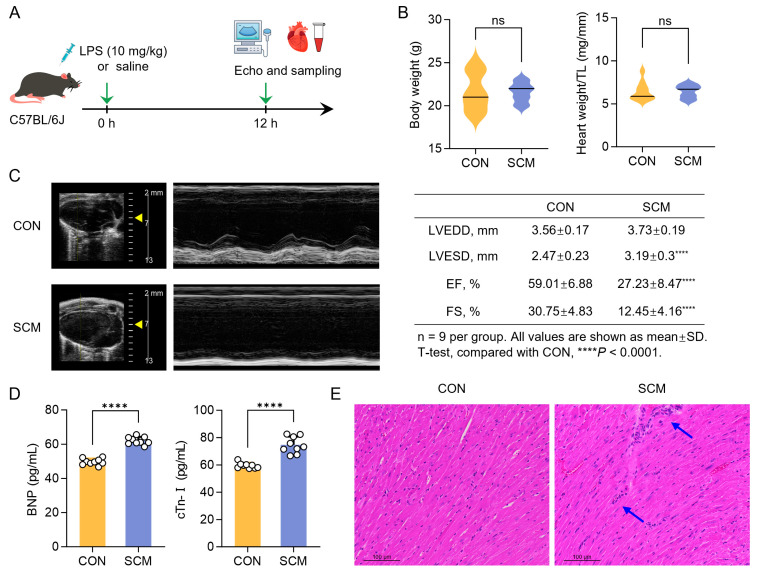
Lipopolysaccharide (LPS)-induced septic cardiomyopathy (SCM) in mice. (**A**) Schematic of SCM mouse model induced by LPS (*n* = 9). (**B**) Body weight and heart weight/tibia length ratio (heart weight/TL) in each group. (**C**) Representative images and data of conventional echocardiography. (**D**) Serum levels of brain natriuretic peptide (BNP) and cardiac troponin I (cTn-I). (**E**) Histological examination of mouse hearts with hematoxylin-eosin (H&E) staining (*n* = 3), blue arrows: inflammatory cell infiltration. Student’s *t*-test, **** *p* < 0.0001, ns: no significant difference.

A single intraperitoneal (*i.p.*) injection of 10 mg/kg LPS was administered to male C57BL/6J mice to induce a model of SCM (Figure 2A). Compared with the control group, no significant differences in body weight or heart weight were observed in the SCM mice (Figure 2B). Echocardiographic analysis revealed that heart function in the SCM group was significantly impaired, as evidenced by the reductions in EF and FS. The left ventricular diameter during the end of systole (LVESD) was significantly increased (Figure 2C). These parameters are crucial indicators of systolic function. Additionally, two key clinical biomarkers (BNP and cTn-I) showed marked elevation in SCM mice (Figure 2D). BNP is released in response to ventricular volume overload and elevated wall stress, while cTn-I is a highly specific marker of myocardial injury. These elevations confirm significant cardiac stress and damage in the SCM group. Histopathological examination of heart tissue using H&E staining revealed clear signs of myocardial injury, including areas of myocardial fiber disarray and the presence of inflammatory cell infiltration (Figure 2E). Taken together, these results demonstrated that the SCM model was successfully established in the LPS-treated mice.

### 2.2. Metabolic Signatures of SCM

**Figure 3:**
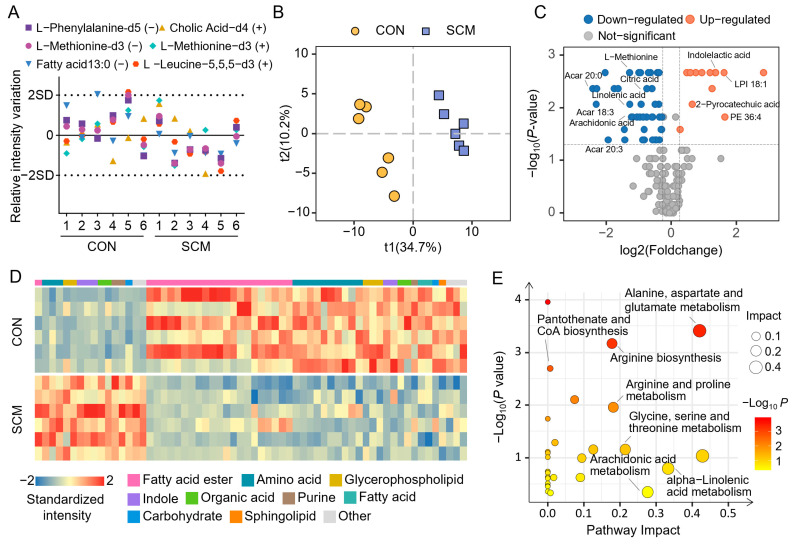
Metabolomics analysis of septic cardiomyopathy (SCM) mouse heart. (**A**) The intensity variation in five internal standards among all samples. (**B**) Principal component analysis (PCA) scatter plot shows metabolic differences in mouse hearts between two groups. (**C**) Volcano plot of metabolites detected in mouse hearts. (**D**) Normalized intensity of 62 differential metabolites. (**E**) Pathways enriched based on the differential metabolites.

We elucidate the global metabolic features of SCM using a UPLC-MS/MS-based pseudo-targeted metabolomics approach that simultaneously semi-quantified more than 259 metabolites (File S1 Data 1). A total of 176 metabolites, including 25 amino acids, 18 fatty acids, 38 fatty acid esters, and 14 organic acids, were detected in the mouse hearts. To ensure the data quality, five isotopic internal standards were added to each sample during preparation. The response values of most internal standards remained within two times the standard deviation, confirming the stability and reproducibility of the analytical method (Figure 3A). The partial least squares discriminant analysis (PLS-DA) scatter plot demonstrated a clear separation between control and SCM samples, confirming that sepsis induced significant alterations in the cardiac metabolic profile (Figure 3B). Using variable importance projection (VIP) >1, absolute fold change >1.2, and *p* < 0.05 (Mann–Whitney U test) as screening criteria, we identified 62 differential metabolites between control and SCM (Figure 3C, File S1 Data 2). As shown in Figure 3D, sepsis led to a marked decrease in most amino acids, fatty acid esters, and glycerophospholipids levels in the heart, while indoles showed significant accumulation. Moreover, the enrichment analysis revealed key metabolic pathways affected, including fatty acid metabolism, amino acid metabolism, and pantothenate biosynthesis (Figure 3E). Therefore, these results suggested that SCM led to profound metabolic alterations, particularly in energy metabolism and lipid handling.

### 2.3. Genetic Signatures of SCM

**Figure 4:**
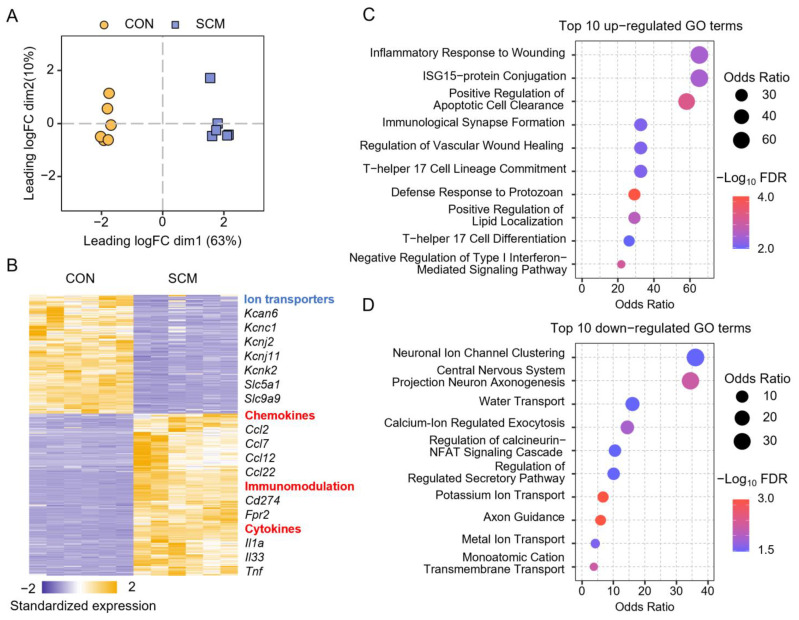
Transcriptomics analysis of septic cardiomyopathy (SCM) mouse heart. (**A**) Multidimensional scaling (MDS) scatter plot shows genetic differences in mouse hearts between two groups. (**B**) Standardized expression of differentially expressed genes (DEGs) in the mouse hearts. (**C**,**D**) Gene ontology (GO) enrichment analysis of 453 upregulated (**C**) and 330 downregulated (**D**) DEGs. The top 10 (highest odds ratio) enriched GO terms of the biological process category are shown.

We also explored the genetic features of SCM using the second-generation sequencing to reveal the presence and quantity of RNA molecules in mouse hearts. After processing the RNA-seq data, we identified 12,683 genes. Multidimensional scaling (MDS) analysis showed a clear separation between control and SCM samples (Figure 4A), indicating a distinct gene expression profile in the hearts of SCM mice, which reflects the extensive transcriptional changes induced by sepsis. Using a cutoff of absolute fold change >2 and a false discovery rate (FDR) < 0.01, a total of 783 differentially expressed genes (DEGs) were screened out (File S1 Data 3). As shown in Figure 4B, compared with the controls, SCM hearts exhibit significantly reduced expression of transporter genes, such as *Kcnj11*, *Kcnk2*, *Slc5a1*, and *Slc9a9*. Conversely, genes involved in cytokine signaling (e.g., *Il1a* and *Il33*), chemokine activity (e.g., *Ccl2* and *Ccl7*), and immune responses (e.g., *Cd274* and *Fpr2*) were significantly upregulated. Gene Ontology (GO) biological process enrichment analysis revealed that upregulated DEGs were primarily involved in pathways that enhance immune responses, such as positive regulation of apoptotic cell clearance and immunological synapse formation, suggesting an inflammatory and immune activation state in SCM (Figure 4C). Meanwhile, downregulated DEGs were enriched in pathways related to ion transport (e.g., potassium ion transport) and muscle contraction (e.g., calcium-ion-regulated exocytosis), highlighting a disruption in cardiac ion homeostasis and contractile function (Figure 4D). Altogether, these findings revealed significant alterations in cardiac gene expression associated with SCM, suggesting the involvement of immune activation and ion disorder in the impairment of heart function.

### 2.4. Identification of SCM-Associated Module

**Figure 5:**
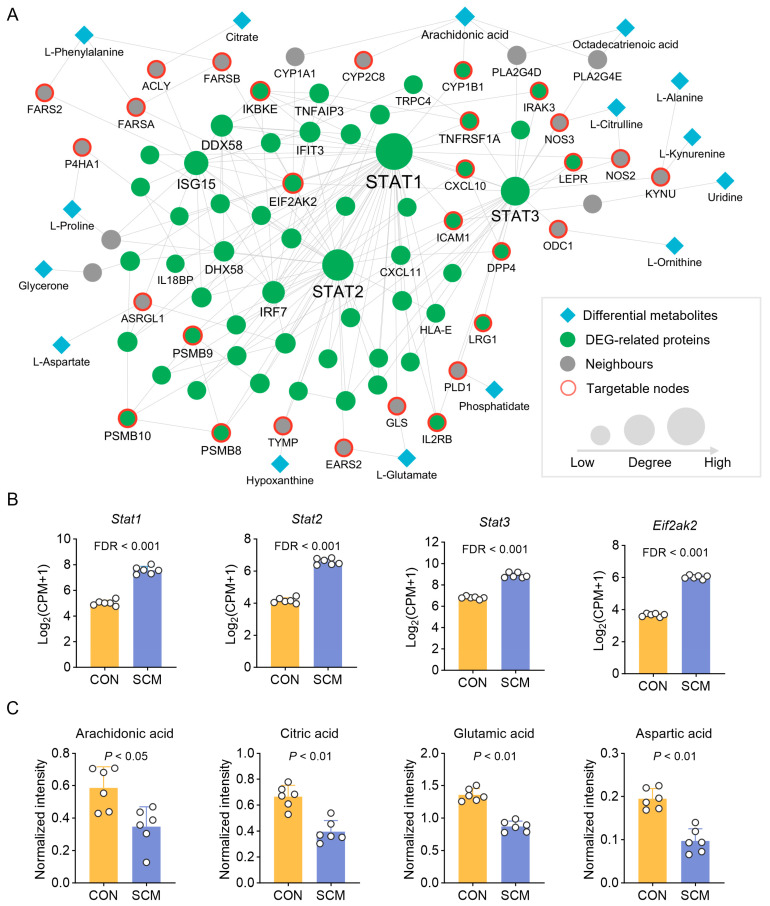
Identification of septic cardiomyopathy (SCM)-associated module. (**A**) A subnetwork of SCM-associated module in the human protein–protein interactome formed by integrating data from metabolomics and transcriptomics analyses. (**B**) Gene expression of *Stat1*, *Stat2*, *Stat3*, and *Eif2ak2* in transcriptomics analysis. False Discovery Rate (FDR) was calculated using moderated t-statistic with the Benjamani–Hochberg procedure. (**C**) Expression of representative metabolites arachidonic acid, citric acid, glutamate acid, and aspartic acid in metabolomics analysis. *p*-value was calculated by using the Mann–Whitney U test.

Previous network medicine studies have demonstrated that disease-associated proteins tend to cluster into connected subgraphs within the human PPI, forming disease modules [22]. These modules have been instrumental in elucidating the mechanisms and discovering therapeutic targets for complex diseases such as cardiovascular disease, Alzheimer’s disease, and cancers [23,24]. Genome-wide Positioning Systems network (GPSnet) is an integrated, network-based methodology that identifies disease modules by incorporating metabolome and transcriptome analyses into the human protein–protein interactome, specifically utilizing the fold changes in DEGs and differential metabolites as node scores and employing the human metabolite–protein network as the background to identify the SCM-associated module [25]. This module consists of 76 proteins, 17 metabolites, and 191 edges (Figure 5A). Notably, signal transducer and activator of transcription (STAT) proteins (e.g., STAT1, STAT2, and STAT3) emerged as central nodes with high connectivity, indicating their significant influence in the SCM-associated module. The STAT protein families are intracellular transcription factors that mediate many aspects of cellular immunity, proliferation, apoptosis, and differentiation [26]. Yu et al.’s study revealed that STAT3 activation plays a crucial role in the generation of reactive oxygen species (ROS) and impedes autophagic flux during septic cardiomyopathy, ultimately resulting in myocardial dysfunction [27]. Peng et al. found that the compound ACT001 rescued mice from septic shock by protecting the cardiovascular system, partly via inhibiting pro-inflammatory cytokine production and down-regulating the JAK-STAT signaling pathway [28]. The evidence indicates that STAT proteins have a significant impact during SCM, and targeting these proteins may offer new therapeutic strategies. Among metabolites, arachidonic acid, citric acid, and several amino acids stood out, suggesting their involvement in the metabolic disturbance associated with SCM. When comparing the levels of these key proteins and metabolites between control and SCM groups, significant alterations were observed (Figure 5B,C). For instance, the expression of *Stat1* was upregulated in the SCM group, while arachidonic acid showed decreased levels. Interestingly, we found that 29 proteins (38.2%) within the SCM-associated module can be targeted by at least one FDA-approved drug. For instance, NOS2, DPP4, NOS3, ODC1, and IL2RB represent the most targetable proteins (Figure S1). This high degree of druggability motivated further drug repurposing studies to explore potential treatments of SCM by directly targeting the disease module.

### 2.5. Network-Based Discovery of Repurposable Drugs for SCM

Given that proteins served as drug targets for a specific disease may also be suitable drug targets for potential anti-infection, we next aimed to identify existing drugs that could be repurposed for SCM therapy. Using a state-of-the-art network proximity measure, which evaluates the physical closeness or logical interconnectedness of nodes in a network, we quantified the relationship between the SCM-associated module and drug targets in the human metabolite–protein network [24]. A drug–target network was constructed by integrating target data for 1930 FDA-approved drugs. To minimize bias, we applied a Z-score metric and a permutation test to assess the proximity between drugs and the SCM. In total, we computationally identified 129 drugs associated with SCM (Z-score < −2.5 and *p* < 0.01, File S1 Data 4). Among these, glutamic acid and pyridoxal phosphate (both metabolic supplements, Z < −8 and *p* < 0.0001) emerged as top candidates, alongside α-tocopherol succinate (an antioxidant, Z < −6 and *p* < 0.0001). Additionally, carfilzomib (a proteasome inhibitor), metreleptin (a leptin analog), and tipiracil (a thymidine phosphorylase inhibitor) also showed strong associations (Z < −5 and *p* < 0.0001), suggesting their potential for SCM treatment.

**Figure 6:**
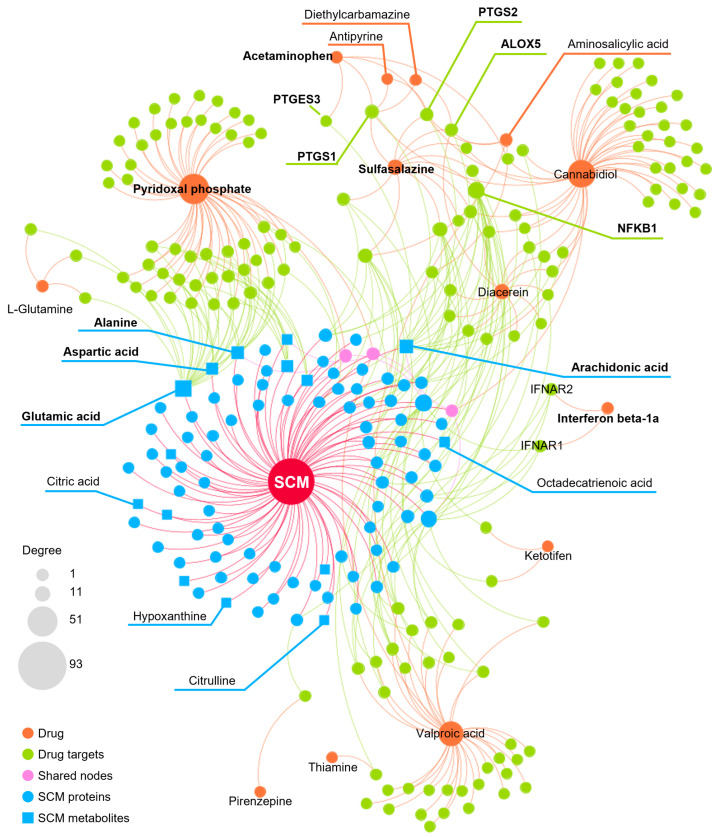
The network connecting 14 FDA-approved drugs and septic cardiomyopathy (SCM).

We further refined our selection to 14 high-confidence repurposable drugs (Figure 6) based on a combination of factors: (i) strong network-predicted associations (a smaller Z-score), (ii) existing literature evidence of anti-infective properties, (iii) drug formulations that are suitable for ICU use, and (iv) lower incidence of adverse effects. Table 1 showcases these selected drugs with their original therapeutic uses and the literature-reported anti-infection evidence. Nearly half (6/14) of these drugs were analgesics (antipyrine and acetaminophen), vitamins (pyridoxal phosphate and thiamine), or anti-inflammatory agents (sulfasalazine and diacerein). Acetaminophen (Z = −2.6 and *p* = 0.003), for example, was predicted to affect key SCM-related proteins such as PTGS1, PTGS2, and PTGES3, and metabolites like arachidonic acid. Notably, the latest clinical trial has shown that acetaminophen, in addition to its pain-relieving and antipyretic properties, reduces the harmful effects of cell-free hemoglobin on the lungs, thereby lowering the risk of organ injury in sepsis patients [29]. Pyridoxal phosphate (Z = −8.3 and *p* < 0.0001), the active form of vitamin B6, is clinically relevant in managing cardiovascular disease and neuropathies due to its role in various metabolic pathways [30]. As shown in Figure 6, pyridoxal phosphate could potentially alleviate SCM by restoring metabolic disruptions, particularly those affecting amino acid metabolism. Previous studies have also demonstrated that vitamin B6 can inhibit LPS-induced ferroptosis and apoptosis in the hearts of mice, suggesting its cardioprotective effects during sepsis [31]. Sulfasalazine (Z = −3.6 and *p* < 0.0001), a salicylate commonly used to treat ulcerative colitis and rheumatoid arthritis, may reduce SCM by targeting inflammation-related proteins like ALOX5 and NFKB1. Additionally, interferon beta-1a (Z = −2.9 and *p* = 0.002), a cytokine used for multiple sclerosis, may attenuate inflammatory processes by modulating pro-inflammatory pathways involved in SCM. Altogether, using network proximity analysis, we identified several FDA-approved drugs as promising candidates for repurposing in SCM.

### 2.6. Acetaminophen and Pyridoxal Phosphate Preserve Heart Function in SCM Mice

**Figure 7:**
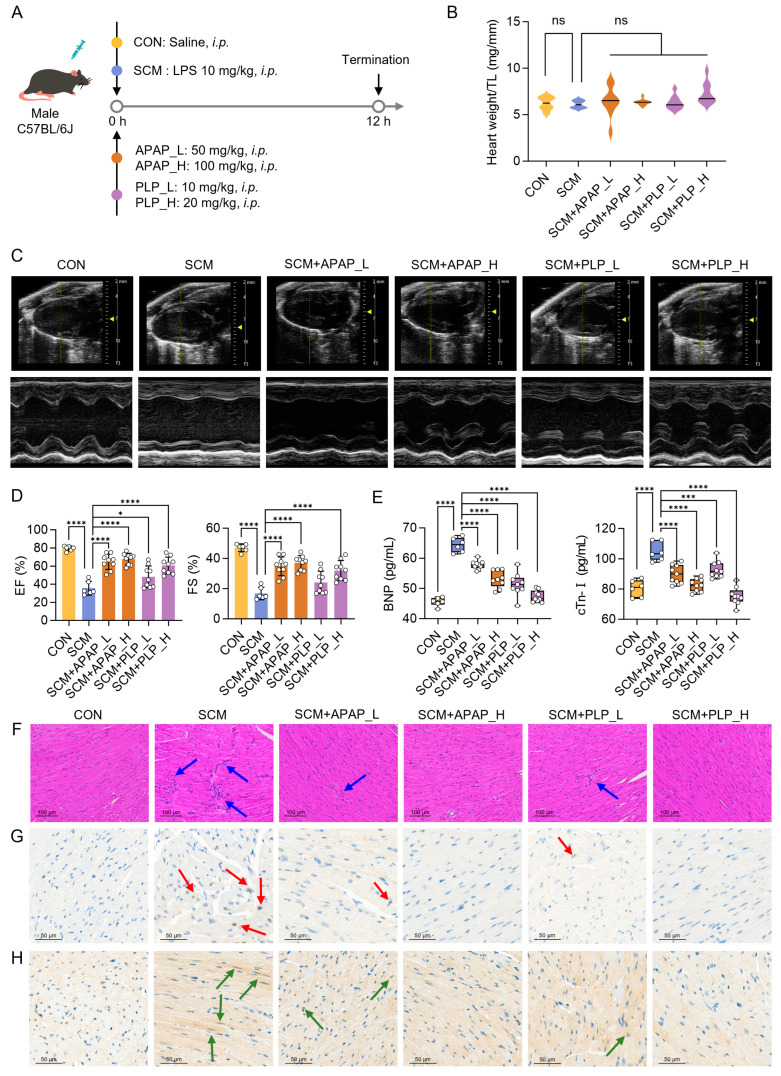
Acetaminophen (APAP) and pyridoxal phosphate (PLP) protect from cardiac injury in septic cardiomyopathy (SCM) mice. (**A**) Schematic of SCM mouse model and intervention. (**B**) Heart weight/tibia length ratio (heart weight/TL) in each group (CON, *n* = 6; SCM, *n* = 6; SCM+APAP_L, *n* = 9; SCM+APAP_H, *n* = 9; SCM+PLP_L, *n* = 9; SCM+PLP_H, *n* = 9). (**C**) Representative images of conventional echocardiography. (**D**) Echocardiographic analysis of mouse heart function. EF: ejection fraction; FS: fractional shortening. (**E**) Serum levels of brain natriuretic peptide (BNP) and cardiac troponin I (cTn-I). (**F**) Histological examination of mouse hearts with hematoxylin-eosin (H&E) staining (*n* = 3), blue arrows: inflammatory cell infiltration. (**G**,**H**) The immunohistochemistry staining of CD45 (**G**) and CD68 (**H**) in mouse hearts, red arrows: CD45-positive cells, green arrows: CD68-positive cells. One-way ANOVA, * *p* < 0.05, *** *p* < 0.001, **** *p* < 0.0001, ns: no significant difference.

We further validated the capabilities of two repurposed drugs, acetaminophen and pyridoxal phosphate, in treating SCM. Mice received a single intraperitoneal injection of LPS (10 mg/kg) to induce SCM, followed by two doses of acetaminophen (50 mg/kg and 100 mg/kg) or pyridoxal phosphate (10 mg/kg and 20 mg/kg) (Figure 7A). The animal experiment ended 12 h after the injection, and no significant differences were observed in body weight or heart weight across the groups (Figure 7B and Figure S2). As expected, echocardiography revealed that both acetaminophen and pyridoxal phosphate significantly improved sepsis-induced cardiac dysfunction, as evidenced by a substantial increase in EF and FS compared to the SCM group (Figure 7C,D). Additionally, serum levels of BNP and cTn-I, which are critical biomarkers of cardiac stress and injury, were significantly reduced in treated groups (Figure 7E). Notably, high-dose pyridoxal phosphate (20 mg/kg) restored the two biomarker levels to those of the control group, indicating near-complete reversal of cardiac injury.

Histopathological examination using H&E staining showed typical signs of SCM in the LPS-treated mice, including cellular disarray and inflammation (Figure 7F). Acetaminophen and pyridoxal phosphate provided clear protection against this damage, as treated mice exhibited reduced inflammation and preservation of myocardial architecture. Immunohistochemical staining for CD68 (a macrophage marker) and CD45 (a general leukocyte marker) showed increased infiltration of immune cells in the hearts of SCM mice (Figure 7G,H). Treatment with acetaminophen or pyridoxal phosphate significantly reduced CD68- and CD45-positive cells, particularly in the high-dose groups, highlighting their anti-inflammatory effects and potential to limit immune-mediated cardiac injury. Taken together, our data demonstrate the beneficial actions of acetaminophen and pyridoxal phosphate for protecting the hearts of SCM mice.

### 2.7. Mechanism-of-Action of Acetaminophen and Pyridoxal Phosphate

**Figure 8:**
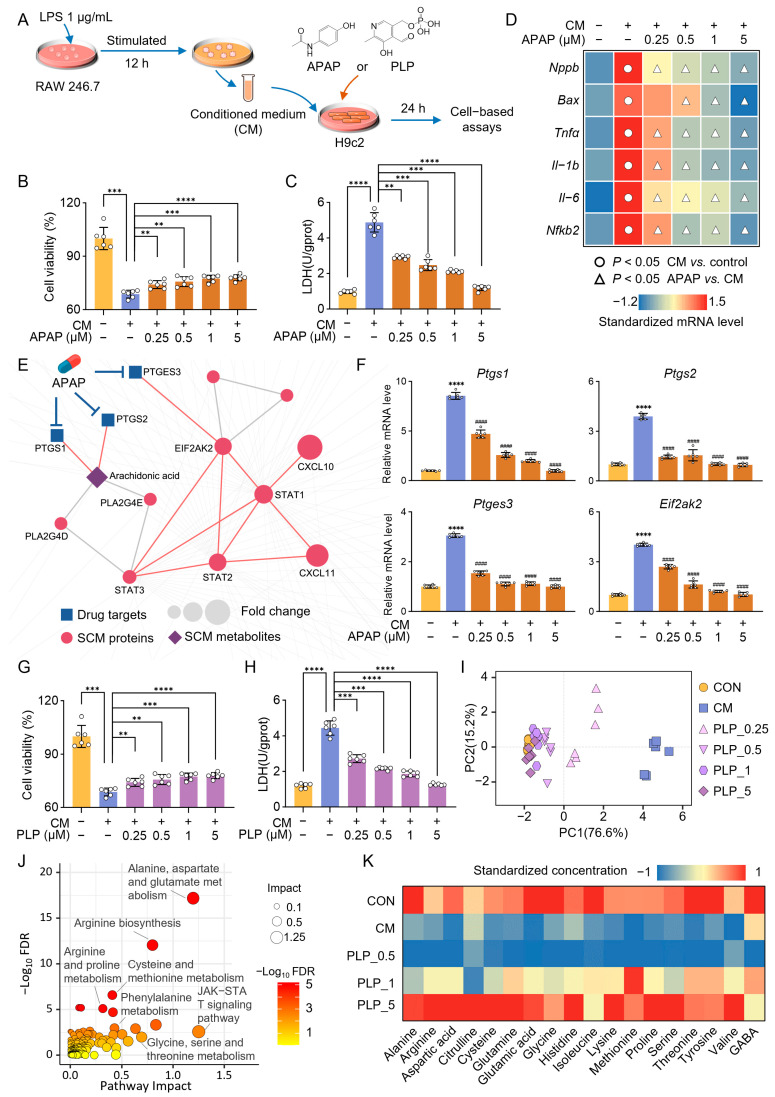
Acetaminophen (APAP) and pyridoxal phosphate (PLP) protect the heart against sepsis by regulating inflammation-related pathways and amino acid metabolism pathways, respectively. (**A**) The workflow of cell experiments. (**B**,**C**) Cell viability (**B**) and lactate dehydrogenase (LDH) activity (**C**) of H9c2 cells following vehicle, conditioned medium (CM), and APAP treatment (*n* = 6). (**D**) Heatmap of normalized expression of genes involved in apoptosis (*Bax*), cardiac injury (*Nppa*), and inflammation (*Tnfα*, *Il-1b*, *Il-6*, and *Nfkb2*) (*n* = 6). (**E**) The highlighted subnetwork shows the inferred mechanism-of-action for APAP’s protective effect in septic cardiomyopathy (SCM). (**F**) Gene expression levels of key targets of APAP in the treatment of SCM (*n* = 6), CM *vs.* control: **** *p* < 0.0001, APAP *vs.* CM: ^####^ *p* < 0.0001. (**G**,**H**) Cell viability (**G**) and cytotoxicity LDH activity (**H**) of H9c2 cells following vehicle, CM, and PLP treatment (*n* = 6). (**I**) Principal component analysis (PCA) scatter plot based on the expression of genes involved in apoptosis (*Bax*), cardiac injury (*Nppa*), and inflammation (*Tnfα*, *Il-1b*, *Il-6*, and *Nfkb2*) (*n* = 6). (**J**) Joint pathway analysis of PLP–SCM interaction network nodes. (**K**) Normalized concentrations of 18 amino acids in H9c2 (*n* = 3). One-way ANOVA, ** *p* < 0.01, *** *p* < 0.001, **** *p* < 0.0001.

We next explored the mechanisms by which acetaminophen and pyridoxal phosphate protect the heart against sepsis using an in vitro model (Figure 8A). The CCK-8 assay measures cell viability by assessing mitochondrial activity, while the LDH assay quantifies cytotoxicity by detecting LDH release from damaged cells. As expected, acetaminophen significantly improved the survival of H9c2 cells (Figure 8B) and reduced LDH release (Figure 8C). PCR analysis further revealed that genes related to apoptosis (*Bax*), cardiac injury (*Nppa*), and inflammation (*Tnfα*, *Il-1b*, *Il-6*, and *Nfkb2*) were significantly elevated in CM-treated cardiomyocytes compared with the control group. Notably, acetaminophen treatment reversed these changes in a dose-dependent manner (Figure 8D). We then integrated drug targets and disease nodes (proteins and metabolites) into the human metabolite–protein network to identify the overlapping pathways between acetaminophen and SCM (Figure 8E). Two key pathways were implicated in acetaminophen’s protective effects: (a) acetaminophen may inhibit PTGS1 and PTGS2, enzymes that convert arachidonic acid into pro-inflammatory prostaglandins, thus reducing inflammation; (b) acetaminophen may modulate inflammatory pathways by inhibiting PTGES3, which affects eukaryotic translation initiation factor 2-alpha kinase 2 (EIF2AK2) signaling. EIF2AK2 is a stress- and infection-activated kinase that promotes inflammation by mediating the activation of the transcription factor NF-κB [32]. Consistent with these network-based predictions, acetaminophen treatment significantly inhibited the expression of *Ptgs1*, *Ptgs2*, and *Ptges3*, as well as *Eif2ak2*, in H9c2 cells (Figure 8F).

Pyridoxal phosphate similarly demonstrated protective effects on H9c2 cardiomyocytes, improving cell survival (Figure 8G) and reducing LDH levels (Figure 8H). Principal component analysis of eight genes related to apoptosis, cardiac injury, and inflammation showed that the cell state treated with pyridoxal phosphate was more similar to the control group compared to the SCM group (Figure 8I). Joint pathway analysis based on the drug–disease interaction network (Figure S3) suggested that pyridoxal phosphate may treat SCM mainly by rescuing metabolic dysfunction, particularly amino acid metabolism (Figure 8J). Given that pyridoxal phosphate is essential for transaminase enzymes, which facilitate the transfer of amino groups between amino acids and α-keto acids, we quantitatively characterized 18 amino acids in H9c2 cells using LC-MS/MS. As shown in Figure 8K, pyridoxal phosphate reverses the significant decreases in amino acid levels caused by SCM, especially glutamic acid, alanine, and glycine. Taken together, our network proximity analysis not only identified the novel protective effects of acetaminophen and pyridoxal phosphate in SCM but also offered testable hypotheses to elucidate the molecular mechanisms underlying their protective actions.

## 3. Discussion

This study presents significant advancements in the treatment of SCM by integrating multi-omics and network analyses, identifying a list of FDA-approved drugs for potential repurposing. The use of network proximity analysis, a cutting-edge method that integrates disease-associated genes, metabolites, and drug targets in the human metabolite–protein interactome, enabled the efficient discovery of 14 candidate drugs. Among them, acetaminophen and pyridoxal phosphate were subjected to in vitro and in vivo validation, demonstrating promising therapeutic effects. In contrast to traditional drug discovery methods [33], which are often time-consuming and costly, this computational approach dramatically accelerates the identification of repurposable drugs.

Mechanistic studies further indicated that acetaminophen reduces inflammation by inhibiting key enzymes in the arachidonic acid-prostaglandin pathway, such as PTGS1 and PTGS2, while pyridoxal phosphate acts on amino acid metabolism, particularly reversing the depletion of essential amino acids like glutamate and alanine. The prostaglandins participate in the pathogenesis of hemodynamic collapse, organ failure, and overwhelming inflammation that characterize severe sepsis and shock. Inhibiting prostaglandin synthesis can be beneficial in improving the overall state of sepsis [34,35]. In addition, acetaminophen has already been demonstrated to reduce organ injury in sepsis patients by blocking the harmful effects of cell-free hemoglobin, offering a dual therapeutic effect in SCM [29]. Pyridoxal phosphate has established roles in metabolic diseases, making it a particularly attractive candidate for addressing the metabolic dysregulation observed in SCM [36]. Both drugs are inexpensive, widely available, and have well-documented safety profiles, which positions them as practical candidates for further clinical research in SCM. The evidence generated from this study strengthens the rationale for advancing these drugs to clinical trials, as they could provide a safety and effective treatment option for SCM.

Moreover, several other FDA-approved drugs identified in this study show potential for treating SCM. Among these, the antiparasitic drug diethylcarbamazine and the antituberculosis drug aminosalicylic acid stand out due to their potential mechanisms of action. Both drugs are known to modulate arachidonic acid metabolism and inflammatory signaling pathways, suggesting that they may also exert anti-inflammatory effects in SCM [37,38]. Diethylcarbamazine, for instance, has been shown to inhibit lipoxygenase, an enzyme involved in leukotriene synthesis, while aminosalicylic acid interferes with pro-inflammatory cytokine production. However, further investigations are warranted to validate these drugs in SCM models.

The network proximity analysis identifies drugs based on their proximity to disease modules within the background network, with a Z-score less than 0 indicating potential interaction with the disease. Notably, this proximity does not inherently indicate that the drug has a therapeutic effect; it could also be a toxic effect. For example, phenylalanine, an essential aromatic amino acid that is a precursor of melanin and dopamine, is commonly used as a component of total parenteral nutrition [39]. We observed that phenylalanine had a short distance to the SCM module (Z = −6.4 and *p* < 0.0001), and its level was significantly elevated in the hearts of SCM mice. Previous studies have suggested that elevated level of phenylalanine was associated with pathological inflammation [40,41]. Mechanistically, treating SCM patients with phenylalanine may exacerbate the condition. This example highlights the need for cautious interpretation of network-derived results, emphasizing that biological and clinical knowledge must be integrated to determine the therapeutic relevance of the identified drugs.

This study has some limitations that should be addressed in future work. First, the multi-omics approach used here provides a comprehensive overview of metabolic and gene alterations associated with SCM at the tissue level. However, single-cell analysis would offer a more precise understanding of the cellular heterogeneity within the heart [42,43]. Incorporating single-cell technologies could refine the SCM-associated modules and provide deeper insights into the molecular drivers of SCM. Second, while we validated the efficacy of acetaminophen and pyridoxal phosphate in animal models and cardiomyocyte cultures, clinical data supporting their use in SCM patients are still lacking. Future studies should focus on conducting randomized controlled trials to confirm the safety and efficacy of these drugs in sepsis patients with cardiac dysfunction. Finally, it is essential to consider that SCM is a complex, immune-mediated disease involving multiple molecular targets and pathways. A single drug may not be sufficient to fully address the underlying pathophysiology. Combining repositioned drugs, such as acetaminophen and pyridoxal phosphate, may provide a synergistic effect that enhances their therapeutic efficacy.

## 4. Materials and Methods

### 4.1. Materials

Lipopolysaccharide (LPS, from Escherichia coli O55:B5) was purchased from Sigma-Aldrich (St. Louis, MO, USA). Pyridoxal phosphate and acetaminophen were purchased from Aladdin (Shanghai, China). Other commercial standards, including amino acids and isotope internal standards, are listed in Table S1. The LC-MS grade methanol and acetonitrile were bought from Merck (Darmstadt, Germany). Water was purified with a Milli-Q system (Millipore Corporation, Billerica, MA, USA).

### 4.2. Animals

All animal procedures complied with the Guide for the Care and Use of Laboratory Animals and were approved by the Animal Ethics Committee of China Pharmaceutical University (Ethical code: 2024-01-018). Male C57BL/6J mice (7–8 weeks old) were procured from Jiangsu Huachuang Sino PharmaTech Co., Ltd. (Taizhou, Jiangsu, China). This study involved two distinct animal experiments. In the first experiment, 18 male C57BL/6J mice were randomly assigned into two groups (*n* = 9 per group, Figure 2A). Mice received an intraperitoneal (*i.p.*) injection of LPS (10 mg/kg) to induce SCM over a period of 12 h. The healthy controls were administered an equivalent volume of saline. In the second experiment, mice were randomly divided into six groups (*n* = 6–9 per group, Figure 7A). Following LPS injection, animals were treated immediately with either low or high doses of acetaminophen (50 or 100 mg/kg) or pyridoxal phosphate (10 or 20 mg/kg). Echocardiographic assessments were conducted 12 h post-LPS injection. Subsequently, serum samples were collected via retrobulbar plexus puncture under light isoflurane anesthesia and stored at –80 °C. Euthanasia was performed by administering an overdose of isoflurane, followed by cervical dislocation. Heart tissues were harvested, weighed, and promptly frozen in liquid nitrogen for future analyses.

### 4.3. Cell Culture and Treatment

Rat heart H9c2 myoblasts were obtained from the Type Culture Collection Cell Bank (Chinese Academy of Sciences Committee, Shanghai, China), and RAW 246.7 was purchased from the American Type Culture Collection (ATCC, Manassas, VA, USA). Cells were cultured in Dulbecco’s modified Eagle’s medium (DMEM) with 10% fetal bovine serum (FBS) and 1% penicillin-streptomycin (Beyotime, Shanghai, China) at 37 °C. RAW 264.7 cells were treated with LPS (1 µg/mL) to produce the conditioned medium, which was then used to induce damage in H9c2 cells. Acetaminophen or pyridoxal phosphate was applied to these H9c2 cells, and their efficacy was evaluated using the cell counting kit-8 (CCK-8) and cytotoxicity lactate dehydrogenase (LDH) assay kit.

### 4.4. Metabolomics Analysis

We performed the pseudo-targeted metabolomics analysis based on a reported protocol with slight modification [44]. Briefly, 40 mg of heart tissue was homogenized with 400 μL of an ice-cold methanol/water solution (80/20, *v*/*v*). A 100 μL aliquot of tissue homogenate was added with 400 μL acetonitrile containing five internal standards (L-phenylalanine-d5, cholic acid-d4, L-methionine-d3, fatty acid13:0, and L-leucine-d3). Internal standards were used to correct for variability in sample processing and analysis. This mixture was then vortexed and centrifuged. Subsequently, 450 μL of the supernatant was dried under nitrogen at 37 °C, reconstituted with 60 µL of water/methanol (90:10, *v*/*v*), and finally injected into a Shimadzu LCMS-8060 system (Shimadzu, Tokyo, Japan) for analysis.

Chromatographic separation was carried out on an XSelect HSS T3 column (2.1 × 100 mm, 2.5 μm; Waters, Milford, MA, USA) at a flow rate of 0.35 mL/min and a temperature of 40 °C. The gradient elution employed a mobile phase consisting of (A) 6.5 mM ammonium acetate in water and (B) methanol. Mass spectrometric detection was performed using an electrospray ionization source, operating in both positive and negative ion modes with multiple-reaction monitoring. There were 6 biological replicates to ensure statistical power, reduce false positives, and increase the reliability of the results. The details are given in the Supplementary Methods.

### 4.5. RNA Sequencing

Samples were submitted to Shanghai Biotechnology Corporation (SHBIO, Shanghai, China) for RNA-seq. The sequencing platform used was Illumina NovaSeq6000 (Illumina Inc., San Diego, CA, USA) in the PE150 mode (Pair-end 150bp). Details of the experimental protocol and data processing are provided in the Supplementary Methods.

### 4.6. Building the Human Metabolite–Protein Network

We initially retrieved the human PPI from a comprehensive resource that compiled interactions from 21 bioinformatics and systems biology databases [45]. This network contains 327,924 interactions (edges) among 18,505 unique proteins (nodes), representing the most complete human protein–protein interactome (PPI) to date. However, human PPI includes only proteins and lack metabolites. We then collected the genomic and chemical information from the Kyoto Encyclopedia of Genes and Genomes (KEGG) database (Version 110.1) and built a human metabolic network that contains 16,994 edges, linking 2220 unique metabolites and 2017 enzymes [46]. Integrating these two networks yielded a human metabolite–protein network with 20,759 nodes and 339,157 edges.

### 4.7. Construction of Disease Module

GPSnet algorithm starts with a randomly selected node (protein or metabolite) as the seed module [25]. For each candidate node (first order neighbors of current seed module), we calculated the updated module score $Z_{m+1}(i)$ (Equation (1)) and the *p*-value of connectivity significance P(i) (Equation (2)).

(1) $$Z_{m+1}\left(i\right)=\frac{\left(s\left(i\right)-\mu \right)+Z_{m}\sqrt{m}}{\sqrt{m+1}}$$

(2) $$P\left(i\right)=\sum_{k=k_{m}}^{k_{i}}\frac{\left(\begin{array}{c} m\\ k \end{array}\right)\left(\begin{array}{c} N-m\\ k_{i}-k \end{array}\right)}{\left(\begin{array}{c} N\\ k_{i} \end{array}\right)}$$

where s(i) is the score of node i, μ denotes the average node score of all nodes in the background network, m represents the number of nodes in module M, N is the number of nodes in the whole background network, $k_{i}$ is the degree of node i, and $k_{m}$ is the number of gene i’s neighbors that belong to the module M.

The node i, with $Z_{m+1}\left(i\right)>Z_{m}$ and $P\left(i\right)<0.01$, will be included in the growing module with probability $\frac{s(i)}{\sum_{i\in n}s(i)}$, where n is the set of the candidate nodes. We repeated these steps until no more nodes could be added. In this study, we built 100,000 raw modules and ranked them in decreasing order of their scores. In total, 5% of the most frequent nodes in the top 1% of modules were selected to assemble the final SCM module.

### 4.8. Network Proximity Measure

To quantify the relationship between a drug and SCM in the human metabolite–protein network, we adopted the shortest-based network proximity measure as below [24].

(3) $$d\left(T,S\right)=\frac{1}{\left|\left|T\right|\right|}\sum_{t\in T}{min}_{s\in S}d(t,s)$$

where d(t,s) is the shortest path length between a target (t) of a drug (T) and a node (s) of the CV module (S). To evaluate whether such proximity was significant, we performed the permutation test by randomly selecting a group of nodes of the same size and degree distribution as the original drug targets in the network. The network proximity was normalized to Z-score ($z=\frac{d-µ}{\sigma }$), where µ and σ are the mean and standard deviation of the 1000 random experiments. *p*-value was also calculated based on the permutation test results. A total of 1930 FDA-approved drugs, as well as their targets, were collected from the DrugBank database (Version 5.1.12). Drugs with z-score < −2.5 and *p* < 0.01 were considered as candidates for the treatment of SCM. The networks were visualized using Gephi 0.10.1 (https://gephi.org/) accessed on 1 July 2024 or Cytoscape 3.10.2 (https://cytoscape.org/) accessed on 1 July 2024.

### 4.9. BNP and cTn-I Test

BNP and cTn-I levels were measured using a commercially available enzyme-linked immunosorbent assay kit according to the manufacturer’s specifications (Nanjing SenBeiJia Biological Technology Co., Ltd., Nanjing, China).

### 4.10. Histologic Analysis

Heart tissues were fixed in 4% paraformaldehyde and embedded in paraffin. Sections were cut at 2-μm thick from the paraffin blocks and stained with hematoxylin and eosin (H&E) according to standard procedures. Histological structures in images were identified using Aperio ImageScope v12.3 software (Leica Biosystems, Vista, CA, USA).

### 4.11. Immunohistochemistry Analysis

Immunohistochemistry (IHC) analysis was conducted on cardiac paraffin sections to assess the expression of CD45 and CD68 in cardiac tissues. After the deparaffinization and antigen retrieval process, slides were incubated with primary antibodies (anti-CD45 and anti-CD68) overnight at 4 °C, then incubated with biotinylated anti-rabbit IgG secondary antibodies. The 3,3′-diaminobenzidine (DAB) was used to reveal the immunohistochemical reaction. The outcomes were scrutinized and captured using Aperio ImageScope v12.3 software (Leica Biosystems, Vista, CA, USA).

### 4.12. Echocardiography

Echocardiography was conducted using the Vevo^®^ 3100LT high-resolution ultrasound system (Fujifilm VisualSonics, Toronto, ON, Canada), employing an ultrahigh-frequency MX400 transducer (30 MHz). An operator, blinded to the study groups, performed the measurements. Mice were anesthetized with isoflurane (3% for induction and 1.5% for maintenance) and placed on a 37 °C heated platform. Parameters, such as left ventricular fractional shortening (FS) and ejection fraction (EF), were calculated by Vevo LAB 3.2.6 software (Fujifilm VisualSonics).

### 4.13. Quantitative Real-Time Polymerase Chain Reaction (RT-PCR)

Total RNA was extracted from heart tissue using the TRIZOL reagent (Keygen Biotech, Nanjing, China), and cDNA was synthesized using the ReverTra Ace^®^ qPCR RT Master Mix (TOYOBO, Shanghai, China), according to the provided protocols. RT-PCR was performed using the ChamQ SYBR green qPCR Master Mix (Vazyme, Nanjing, China) on a LightCycler 96 Instrument (Roche, Mannheim, Germany). The primer sequences are listed in Table S2.

### 4.14. Quantification of Amino Acids

The quantification of amino acids was performed using the HILIC-MS/MS method established in our previous study [47]. Briefly, H9c2 cells were washed with PBS on ice, scraped from the plates into 1 mL methanol, and quenched in liquid nitrogen and under ultrasonic. The cell extract was dried under nitrogen at 37 °C, reconstituted with 100 µL of acetonitrile/water (85:15, *v*/*v*), and finally injected into a Shimadzu LCMS-8060 system (Shimadzu, Tokyo, Japan) for analysis. The separation was performed on an XBridge BEH Amide column (2.1 × 100 mm, 2.5 μm, Waters, Milford, MA, USA) column with a 0.3 mL/min flow rate at 35 °C. The gradient elution involved a mobile phase consisting of (A) 5 mM ammonium acetate in water with formic acid adjusting pH to 3.0 and (B) acetonitrile. The MS detection was achieved using an ESI source in negative ion mode. The details are given in the Supplementary Methods.

### 4.15. Statistical Analysis

The metabolomic and transcriptomic data were uploaded into R-Studio software (version 4.3.3) for multivariate statistical analysis, including principal component analysis (PCA), partial least-squares discriminant analysis (PLS-DA), and multidimensional scaling (MDS). Comparisons between two groups were performed using Student’s *t*-test, and comparisons between multiple groups were performed using one-way analysis of variance (ANOVA). Plots and statistical tests were carried out using GraphPad Prism 10 (GraphPad Software, Inc., San Diego, CA, USA). Statistical values which achieved a *p*-value of <0.05 were considered statistically significant. Gene ontology (GO) functional annotation was performed using the website tool Enrichr (https://maayanlab.cloud/Enrichr/) accessed on 12 July 2024, and joint pathway analysis was performed using MetaboAnalyst 6.0 (https://www.metaboanalyst.ca/MetaboAnalyst/) accessed on 15 July 2024.

## 5. Conclusions

In summary, this study successfully applied a network medicine approach, integrating metabolomics and transcriptomics analyses, to identify and validate potential therapeutic candidates for SCM. We identified a total of 14 FDA-approved drugs with potential for repurposing. Importantly, acetaminophen and pyridoxal phosphate were shown to significantly improve cardiac function and reduce biomarkers of heart injury in SCM, both in vitro and in vivo. Mechanistic studies revealed that these drugs exert their effects by modulating key pathways involved in inflammation and amino acid metabolism. These findings lay the groundwork for novel therapeutic strategies aimed at improving outcomes in patients with SCM.

## Figures and Tables

**Table 1:** Information of 14 high-confidence repurposable drugs.

No	Drug Name	DrugBank ID	Structure	Z-Score	*p* Value	Known Indications
1	Pyridoxal phosphate	DB00114	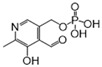	−8.33	0.000	Vitamin supplementation
2	L-Glutamine	DB00130	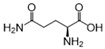	−3.71	0.000	Nutritional supplementation
3	Sulfasalazine	DB00795	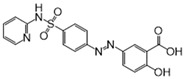	−3.60	0.000	Anti-inflammatory
4	Diacerein	DB11994	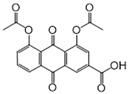	−3.25	0.000	Anti-inflammatory
5	Pirenzepine	DB00670	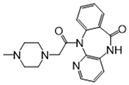	−3.05	0.003	Anti-muscarinic
6	Aminosalicylic acid	DB00233	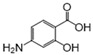	−3.01	0.001	Anti-mycobacteria
7	Interferon beta-1a	DB00060	Protein drug	−2.92	0.002	Immune stimulation
8	Cannabidiol	DB09061	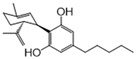	−2.91	0.000	Anti-epileptic
9	Valproic acid	DB00313	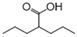	−2.86	0.001	Anti-epileptic
10	Thiamine	DB00152	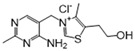	−2.83	0.007	Vitamin supplementation
11	Antipyrine	DB01435	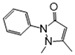	−2.82	0.001	Analgesia
12	Acetaminophen	DB00316	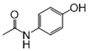	−2.63	0.003	Analgesia
13	Diethylcarbamazine	DB00711	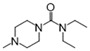	−2.62	0.001	Anthelmintic
14	Ketotifen	DB00920	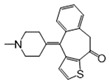	−2.52	0.003	Antihistamines

## Data Availability

The data that support the findings of this study are contained within the article; further inquiries can be directed to the corresponding authors.

## Supplementary Materials

The following supporting information can be downloaded at: https://www.mdpi.com/article/10.3390/ph18010043/s1, Method: Detailed information for pseudo-targeted metabolomics and amino acid determination. Figure S1: Targetable proteins in SCM-associated module. Figure S2: Body weight in each group. Figure S3: A highlighted subnetwork shows the inferred mechanism-of-action for pyridoxal phosphate’s protective effect in septic cardiomyopathy (SCM) network analysis. Table S1: Information on chemical standards. Table S2: Primer sequences used for real-time polymerase chain reaction (RT-PCR). File S1 Data 1: The multiple reaction monitoring (MRM) parameters for pseudo-targeted metabolomics; File S1 Data 2: List of 62 differential metabolites; File S1 Data 3: List of 783 differentially expressed genes (DEGs); File S1 Data 4: List of 129 drugs associated with SCM.
