# Correction: Liu et al. Multi-Omics and Network-Based Drug Repurposing for Septic Cardiomyopathy. *Pharmaceuticals* 2025, *18*, 43

**DOI:** 10.3390/ph18071040

**Published:** 2025-07-14

**Authors:** Pei-Pei Liu, Xin-Yue Yu, Qing-Qing Pan, Jia-Jun Ren, Yu-Xuan Han, Kai Zhang, Yan Wang, Yin Huang, Tao Ban

**Affiliations:** 1 Department of Pharmacology, College of Pharmacy, Harbin Medical University, Harbin 150081, China; 2 Key Laboratory of Drug Quality Control and Pharmacovigilance, Ministry of Education, China Pharmaceutical University, Nanjing 210009, China; 3 Department of Pharmaceutical Analysis, School of Pharmacy, China Pharmaceutical University, Nanjing 210009, China; 4 Department of Critical Care Medicine, Nanjing Drum Tower Hospital, Clinical College, Nanjing Medical University, Nanjing 210008, China; 5 State Key Laboratory of Frigid Zone Cardiovascular Diseases, Ministry of Science and Technology, Harbin Medical University, Harbin 150081, China; 6 Key Laboratory of Cardiovascular Research, Ministry of Education, Harbin Medical University, Harbin 150081, China

## Error in Figure

In the original publication [1], there were two mistakes in Figures 7 and 8 as published. Figure 7G included an incorrect image due to file misplacement. Figure 8G incorrectly displayed the dataset intended for Figure 8B during the data import process. The corrected Figure 7 and Figure 8 appear below. The authors state that the scientific conclusions are unaffected. These corrections were approved by the Academic Editor. The original publication has also been updated.

## Figures and Tables

**Figure 7:**
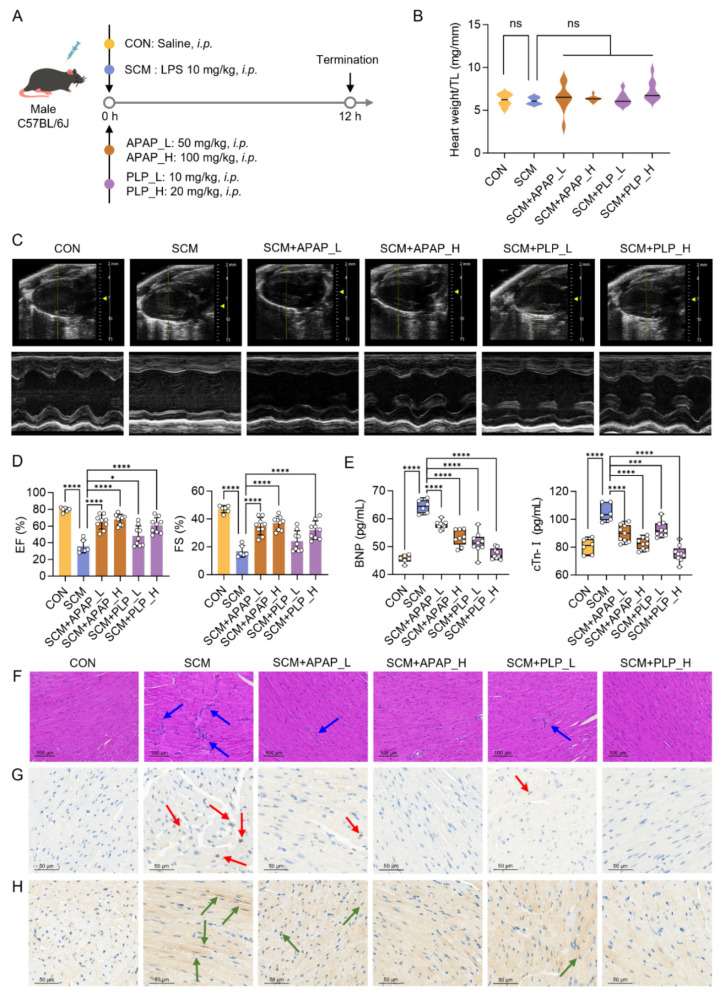
Acetaminophen (APAP) and pyridoxal phosphate (PLP) protect against cardiac injury in septic cardiomyopathy (SCM) mice. (**A**) Schematic of SCM mouse model and intervention; (**B**) heart weight/tibia length ratio (heart weight/TL) in each group (CON, *n* = 6; SCM, *n* = 6; SCM+APAP_L, *n* = 9; SCM+APAP_H, *n* = 9; SCM+PLP_L, *n* = 9; SCM+PLP_H, *n* = 9); (**C**) representative conventional echocardiography images; (**D**) echocardiographic analysis of mouse heart function (EF: ejection fraction; FS: fractional shortening); (**E**) serum levels of brain natriuretic peptide (BNP) and cardiac troponin I (cTn-I); (**F**) histological examination of mouse hearts with hematoxylin–eosin (H&E) staining (*n* = 3) (blue arrows: inflammatory cell infiltration); (**G**,**H**) the immunohistochemical staining of CD45 (**G**) and CD68 (**H**) in mouse hearts (red arrows: CD45-positive cells, green arrows: CD68-positive cells). One-way ANOVA, * *p* < 0.05, *** *p* < 0.001, **** *p* < 0.0001, ns: no significant difference.

**Figure 8:**
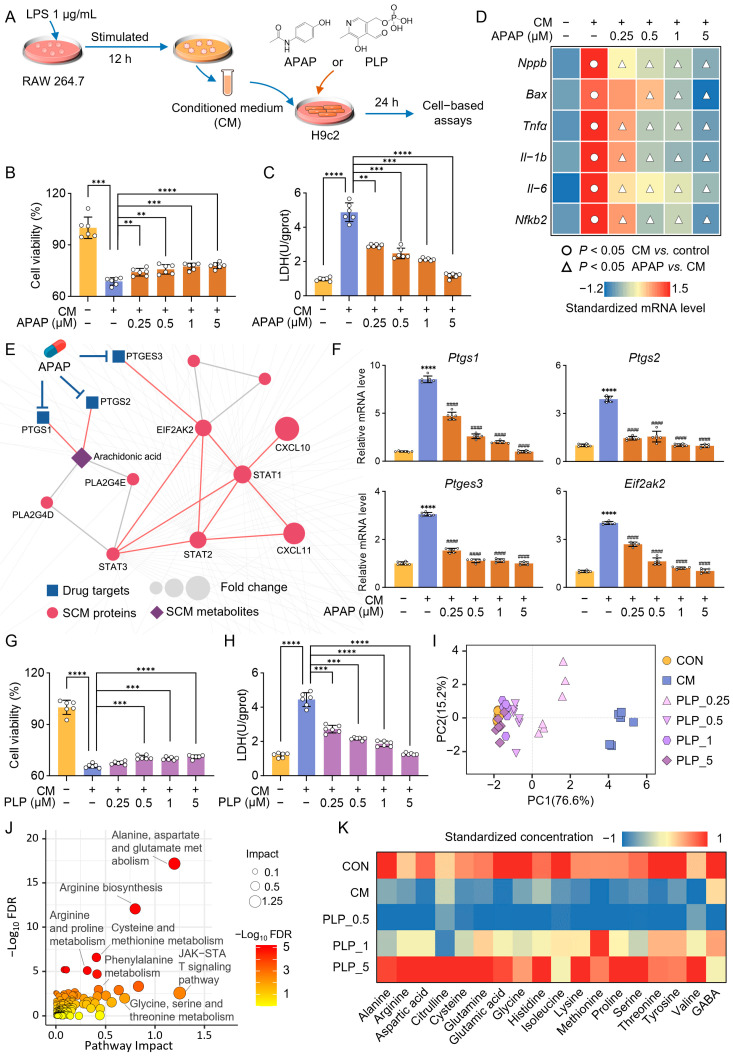
Acetaminophen (APAP) and pyridoxal phosphate (PLP) protect the heart against sepsis by regulating inflammation-related pathways and amino acid metabolism pathways, respectively. (**A**) The workflow of cell experiments. (**B**,**C**) Cell viability (**B**) and lactate dehydrogenase (LDH) activity (**C**) of H9c2 cells following vehicle, conditioned-medium (CM), and APAP treatment (*n* = 6). (**D**) Heatmap of normalized expression of genes involved in apoptosis (*Bax*), cardiac injury (*Nppa*), and inflammation (*Tnfα*, *Il-1b*, *Il-6*, and *Nfkb2*) (*n* = 6). (**E**) The highlighted subnetwork shows the inferred mechanism of action for APAP’s protective effect in septic cardiomyopathy (SCM). (**F**) Gene expression levels of key targets of APAP in the treatment of SCM (*n* = 6), CM vs. control: **** *p* < 0.0001, APAP vs. CM: ^####^ *p* < 0.0001. (**G**,**H**) Cell viability (**G**) and LDH activity (**H**) of H9c2 cells following vehicle, CM, and PLP treatment (*n* = 6). (**I**) Principal component analysis (PCA) scatter plot based on the expression of genes involved in apoptosis (*Bax*), cardiac injury (*Nppa*), and inflammation (*Tnfα*, *Il-1b*, *Il-6*, and *Nfkb2*) (*n* = 6). (**J**) Joint pathway analysis of PLP–SCM interaction network nodes. (**K**) Normalized concentrations of 18 amino acids in H9c2 (*n* = 3). One-way ANOVA, ** *p* < 0.01, *** *p* < 0.001, **** *p* < 0.0001.
