# Supplementary material for: Multi-Omics and Network-Based Drug Repurposing for Septic Cardiomyopathy
Source: Pharmaceuticals (Basel). 2025 Jan 2;18(1):43. doi: 10.3390/ph18010043 (PMC11768530; doi:10.3390/ph18010043)
Supplement: Supplementary file 1 [file pharmaceuticals-18-00043-s001.zip › Supplementary Materials_20241223.pdf]

## Supplementary Materials

## Multi-omics and network-based drug repurposing for septic cardiomyopathy

Peipei Liu[1#], Xinyue Yu[2#], QingQing Pan[3], Jiajun Ren[3], Yuxuan Han[1], Kai Zhang[1], Yan Wang[4], Yin Huang[2,3*], Tao Ban[1,5,6*]

[1] Department of Pharmacology, College of Pharmacy, Harbin Medical University, Harbin, 150081, China.

[2] Key Laboratory of Drug Quality Control and Pharmacovigilance, China Pharmaceutical University, Ministry of Education, Nanjing, 210009, China

[3] Department of Pharmaceutical Analysis, School of Pharmacy, China Pharmaceutical University, Nanjing, 210009, China

[4] Department of Critical Care Medicine, Nanjing Drum Tower Hospital, Clinical College of Nanjing Medical University, Nanjing, 210008, China

[5] State Key Laboratory of Frigid Zone Cardiovascular Diseases, Ministry of Science and Technology, Harbin Medical University, Harbin, 150081, China

[6] Key Laboratory of Cardiovascular Research, Ministry of Education, Harbin Medical University, Harbin, 150081, China

[#] These authors contributed equally to this work.

*Corresponding authors:

Tao Ban, Ph.D., Professor

Email: bantao2000@163.com

Yin Huang, Ph.D., Associate Professor

Email: huangyin@cpu.edu.cn

## Method

### LC-MS/MS analysis for metabolomics

The sample was injected by a Shimadzu LCMS-8060 system (Japan). Reverse phase chromatographic separation was carried out on an XSelect HSS T3 column (2.1 × 100 mm, 2.5 μm; Waters, USA) at a flow rate of 0.35 mL/min and a temperature of 40°C. The gradient elution employed a mobile phase consisting of (A) 6.5 mM ammonium acetate in water and (B) methanol. The elution gradient was performed as follows: 0-1 min：2%B；1-24 min：2-95%B；24-28 min：95%B；28-29 min：95-2%B；29-34 min：2%B. MS detection was performed by an electrospray ionization (ESI) source operated with interface voltage of 4.0 kV in positive mode and -3.0 kV in negative mode. The other main parameters were as follows: nebulizing gas, 3 L/min; drying gas, 15 L/min; heat block temperature, 400°C; desorption line (DL) temperature, 250°C; and ion accumulation time 5 ms. The collision energy (CE) and multiple reaction monitoring (MRM) parameters refer to the literature of Xu et al. (*Nat Protoc.* 2020;15(8):2519-2537.) and were provided in **Appendix Data 1**.

### RNA sequencing

Heart samples were preserved in RNA stabilization reagents (BioYou, China) and submitted to Shanghai Biotechnology Corporation for RNA-seq. Total RNA was extracted using the MJzol Animal RNA Isolation Kit (Majorivd) and purified using RNAClean XP Kit (Beckman Coulter) and RNase-Free DNase Set (QIAGEN) according to standard operating procedures provided by the manufacturer. The mRNA was enriched using VAHTS® mRNA Capure Beads (Human/Mouse/Rat). The mRNA sequencing libraries were constructed using the VAHTS Universal V6 RNA-seq Library Prep Kit for Illumina® (Vazyme) according to standard operating procedures provided by the manufacturer. The sequencing platform used was Illumina

NovaSeq6000, and the sequencing mode adopted PE150 (Pair-end 150bp). Then apply seqtk to filter poor quality reads and trim poor quality bases from samples. Genome mapping of pre-processed reads was performed applying the spliced mapping algorithm of Hisat2 (version: 2.0.4). The level of gene expression is estimated by the number of reads mapped to the gene region. For RNA sequencing data, edge R (ver. 3.34.1) and limma packages (ver. 3.48.3) in R Studio (ver. 4.1.1) were used for processing. The filterByExpr function in the edgeR package was used to delete low-expression genes, and the data were normalized using the Trimmed Mean of M-values (TMM) method. The preprocessed data were then converted to log 2-count per million (log2CPM). Linear models and empirical Bayesian adjustments were used to evaluate differential expression. The empirical Bayesian parameter method was used to calculate P values and other parameters, and the P values for multiple hypothesis testing were corrected by BH (Benjamini and Hochberg).

**HILIC‑MS/MS analysis for quantification of amino acids**

The sample was injected by a Shimadzu LCMS-8060 system (Japan). Chromatographic separation was carried out using an XBridge BEH Amide column (2.1×100 mm, 2.5 μm, Waters, USA) with a flow rate of 0.3 mL/min at 35 °C. The mobile phase consisted of (A) 5 mM ammonium acetate in water with formic acid adjusting pH to 3.0 and (B) acetonitrile. A 17-min elution gradient was performed as follows: during the first 7 min, the proportion of eluent A was linearly increased from 15% to 18%, then a further increase to 50% A reached in 2 min and kept for 2 min. Finally, the initial conditions were recovered in 1 min and maintained for 5 min. The MS detection was conducted using a Shimadzu 8060 triple quadrupole mass spectrometer (Japan) equipped with an electrospray ionization (ESI) source operated in the positive mode with multiple reaction monitoring (MRM). The MS instrument parameters were as follows: spray voltage 4.0

kV, desorption line temperature 250°C, heat block temperature 40°C, nebulizing gas 3.0 L/min, drying gas 10.0 L/min.

The optimized MRM parameters for fatty acids

| Analyte | MW | Precursor ion (m/z) | Product ion (m/z) | CE (eV) |
|---|---|---|---|---|
| Alanine | 89.1 | 90.1 | 44.1 | -13 |
| Arginine | 174.2 | 175.1 | 70.1 | -22 |
| Aspartic acid | 133.1 | 134.6 | 134.6 | -5 |
| Citrulline | 175.2 | 176.1 | 70.1 | -25 |
| Cysteine | 121.2 | 122.4 | 75.9 | -15 |
| Glutamine | 146.1 | 147.1 | 84.1 | -17 |
| Glutamic acid | 147.1 | 148.1 | 84.1 | -16 |
| Glycine | 75.1 | 76.1 | 30 | -12 |
| Histidine | 155.2 | 156.1 | 110.2 | -14 |
| Isoleucine | 131.2 | 132.2 | 86.2 | -11 |
| Lysine | 146.2 | 147.1 | 84.2 | -17 |
| Methionine | 149.2 | 150.1 | 61.1 | -22 |
| Proline | 115.1 | 116.1 | 70.1 | -16 |
| Serine | 105.1 | 106.1 | 60.1 | -12 |
| Threonine | 119 | 120.1 | 74.2 | -11 |
| Tyrosine | 181.2 | 182.1 | 91.1 | -28 |
| Valine | 117.1 | 118.1 | 72 | -11 |
| γ-Aminobutyric acid | 103.1 | 104.1 | 87.1 | -14 |
| L-Leucine-d3 | 134.1 | 135.1 | 89.2 | -11 |
| L-Alanine-d4 | 93.1 | 94.1 | 48.2 | -13 |

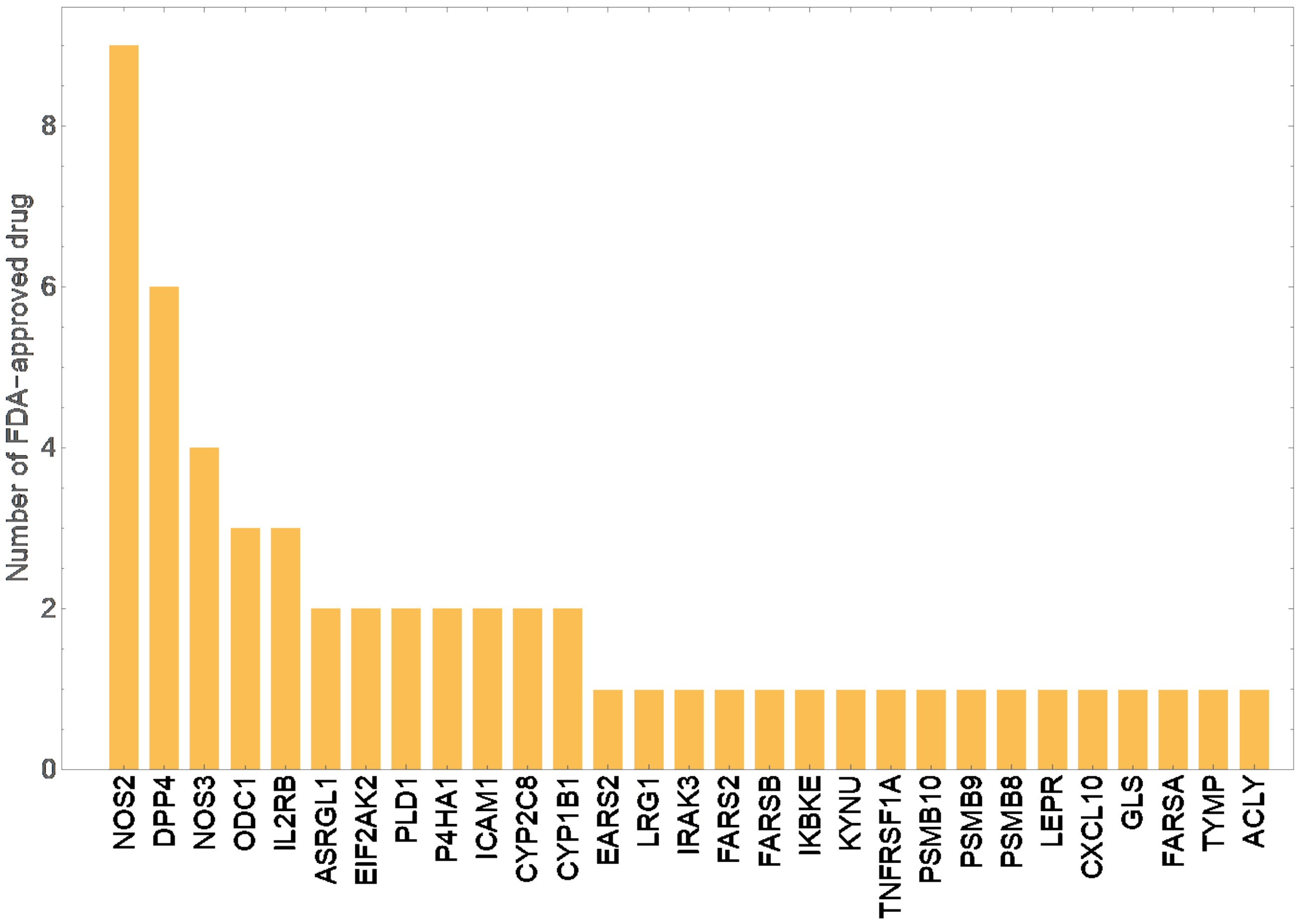


**Figure S1.** Targetable proteins in SCM-associated module.

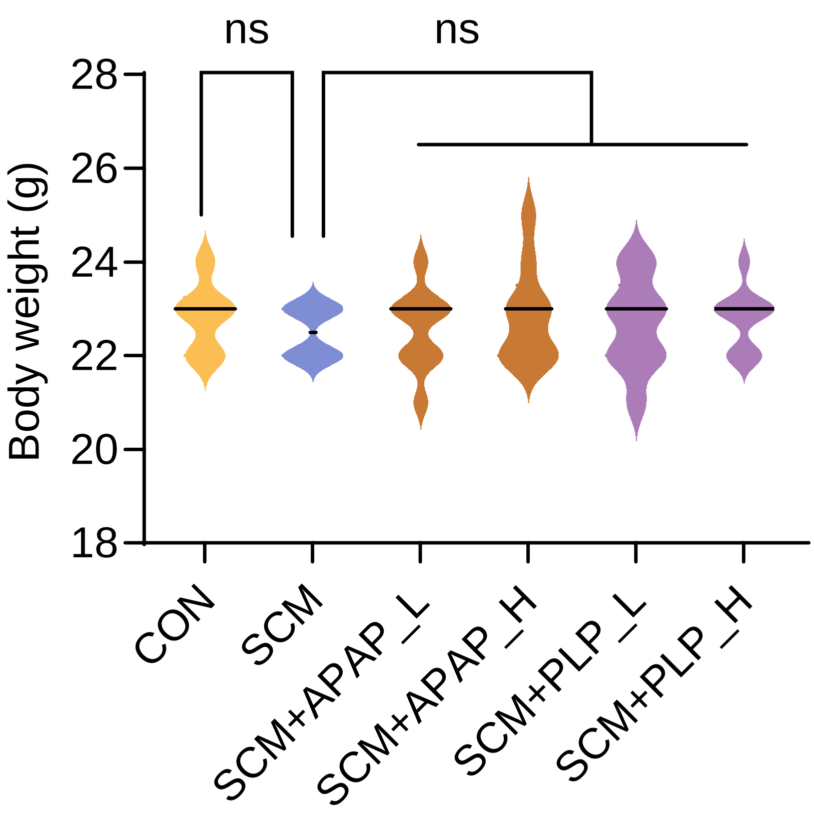


**Figure S2.** Body weight in each group (CON, n = 6; SCM, n = 6; SCM+APAP_L, n = 9; SCM+APAP_H, n = 9; SCM+PLP_L, n = 9; SCM+PLP_H, n = 9). One-way ANOVA, ns: no significant difference.

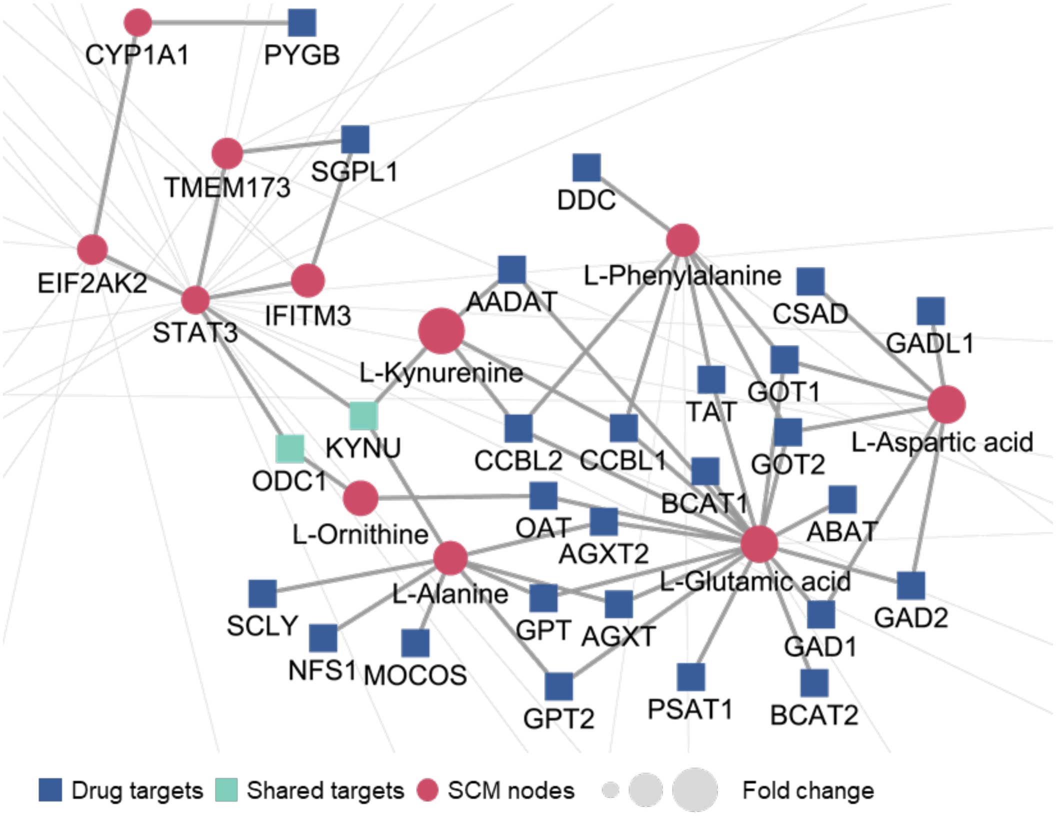


**Figure S3.** A highlighted subnetwork shows the inferred mechanism-of-action for pyridoxal phosphate's protective effect in septic cardiomyopathy (SCM) network analysis.

**Table S1. Information of chemical standards.**

| REAGENT or RESOURCE | SOURCE | IDENTIFIER |
|---|---|---|
| L-Phenylalanine-d5 | Macklin | Cat# L874704 |
| Cholic Acid-d4 | IsoReag | Cat# IR-14894 |
| L-Methionine-d3 | Sigma-Aldrich | Cat# 300616 |
| Fatty acid13:0 | Aladdin | Cat# T114032 |
| L-Leucine-d3 | Sigma-Aldrich | Cat# 486825 |
| L-Alanine-d4 | Cambridge Isotope Laboratories | Cat# DLM-250-1 |
| L-Alanine | Sigma-Aldrich | Cat# A7627 |
| L-Arginine | Aladdin | Cat# A137768 |
| L-Aspartic acid | Aladdin | Cat# A108860 |
| L-Citrulline | Sigma-Aldrich | Cat# 1133842 |
| L-Cysteine | Sigma-Aldrich | Cat# 95437 |
| L-Glutamine | Aladdin | Cat# G105425 |
| L-Glutamic acid | Sigma-Aldrich | Cat# 49449 |
| Glycine | Sigma-Aldrich | Cat# 1295800 |
| L-Histidine | Sigma-Aldrich | Cat# H8000 |
| L-Isoleucine | Sigma-Aldrich | Cat# 58879 |
| L-Lysine | Sigma-Aldrich | Cat# L5501 |
| L-Methionine | Sigma-Aldrich | Cat# M0960000 |
| L-Proline | Aladdin | Cat# P120032 |
| L-Serine | Aladdin | Cat# S137888 |
| L-Threonine | Sigma-Aldrich | Cat# T8625 |
| L-Tyrosine | Sigma-Aldrich | Cat# 93829 |
| L-Valine | Sigma-Aldrich | Cat# 94619 |
| γ-Aminobutyric acid | Aladdin | Cat# A104200 |

**Table S2. Primer sequences used for real-time polymerase chain reaction (RT-PCR).**

| Gene | Forward Primer | Reverse Primer |
|---|---|---|
| *Nppb* | TATAAAAGGCAGAGGCACCG | AATCATCTGGGACAGCACCT |
| *Bax* | CAGTTGAAGTTGCCATCAGC | CAGTTGAAGTTACCATCAGC |
| *Tnfα* | AGCATGATCCGAGATGTGGAA | TAGACAGAAGAGCGTGGTGGC |
| *Il-1b* | CACCTCTCAAGCAGAGCACAG | GGGTTCCATGGTGAAGTCAAC |
| *Il-6* | GTTGCCTTCTTGGGACTGATG | ATACTGGTCTGTTGTGGGTGGT |
| *Nfkb2* | GATCTCGACCTCCACCGGAT | TTCCCAGAGTTTCAGACGCC |
| *Ptgs1* | GTTTCCCCTGCTGCTGCTC | GGCTGGGGATAAGGTTGGAC |
| *Ptgs2* | CTGAAGCCCACCCCAAACAC | TGGGAGTTGGGCAGTCATCA |
| *Ptges3* | GCCCTCTGCCCCGTTCAC | GCTTTGCCCTTTCCTTTGTTA |
| *Eif2ak2* | CGAGAACAGCACAAACGGTG | GCCTTGTCCTCTTGACTCCG |
| *Stat1* | AAGCACCAGAACCGATGGAG | AGCCTCGAGACAGTGCAATC |
| *Stat2* | AAGGGGTGGACCTGCAGAAT | TCACCAGCAGTCTTGTTCGG |
| *Stat3* | GCTTCTCCTTCTGGGTCTGGC | CCTCCTTCTTTGCTGCTTTCACT |
| *Cxcl10* | GGGATCCCTCTCGCAAGAA | CTCAGCGTCTGTTCATGGAAGT |
| *Cxcl11* | GGTTCCAGGCTTCGTTATGT | CTTTGTCACAGCCGTTACTC |
| *Gapdh* | ATTGTCAGCAATGCATCCTG | ATGGACTGTGGTCATGAGCC |
